# The Outbreaks of Acute Encephalitis Syndrome in Uttar Pradesh, India (1978–2020) and Its Effective Management: A Remarkable Public Health Success Story

**DOI:** 10.3389/fpubh.2021.793268

**Published:** 2022-02-09

**Authors:** Neha Srivastava, Hirawati Deval, Mahima Mittal, Rajni Kant, Vijay P. Bondre

**Affiliations:** ^1^ICMR-Regional Medical Research Centre, Gorakhpur, India; ^2^Department of Pediatrics, All India Institute of Medical Sciences, Gorakhpur, India; ^3^Encephalitis Group, National Institute of Virology, Pune, India

**Keywords:** encephalitis, diagnosis, outbreaks, preventive measures, control strategies, disease management

## Abstract

**Introduction:**

Acute encephalitis syndrome (AES) is a major public health enigma in India and the world. Uttar Pradesh (UP) is witnessing recurrent and extensive seasonal AES outbreaks since 1978. Government of India and UP state government have devised various mitigation measures to reduce AES burden and AES associated mortality, morbidity and disability in Uttar Pradesh. The aim of this study was to describe the public health measures taken in order to control seasonal outbreaks of AES in UP between 1978 and 2020.

**Methods:**

We used literature review as a method of analysis, including the Indian government policy documents. This review utilized search engines such as PubMed, Google Scholar, Research Gate, Cochrane, Medline to retrieve articles and information using strategic keywords related to Acute Encephalitis Syndrome. Data was also collected from progress reports of government schemes and websites of Indian Council of Medical Research (ICMR), National Vector Borne Disease Control Programme (NVBDCP) and Integrated Disease Surveillance Programmes (IDSP).

**Results:**

The incidence of AES cases in UP have declined from 18.2 per million population during 2005-2009 to 15 per million population during 2015-2019 [CI 12.6–20.6, *P*-value < 0.001] and case fatality rate (CFR) reduced from 33% during 1980-1984 to 12.6% during 2015-2019 [CI 17.4–30.98, *P*-value < 0.001]. AES incidence was 9 (2019) and 7 (2020) cases per million populations respectively and CFR was 5.8% (2019) and 5% (2020). This decline was likely due to active surveillance programs identifying aetiological agents and risk factors of AES cases. The identified etiologies of AES include Japanese encephalitis virus (5–20%), Enterovirus (0.1–33%), Orientia tsutsugamushi (45–60%) and other viral (0.2–4.2%), bacterial (0–5%) and Rickettsial (0.5–2%) causes. The aggressive immunization programs against Japanese encephalitis with vaccination coverage of 72.3% in UP helped in declining of JE cases in the region. The presumptive treatment of febrile cases with empirical Doxycycline and Azithromycin (EDA) caused decline in Scrub Typhus-AES cases. Decrease in incidence of vector borne diseases (Malaria, Dengue, Japanese Encephalitis and Kala Azar) i.e., 39.6/100,000 population in 2010 to 18/100,000 population in 2017 is highlighting the impact of vector control interventions. Strengthening healthcare infrastructure in BRD medical college and establishment of Encephalitis Treatment Centre (ETC) at peripheral health centres and emergency ambulance services (Dial 108) reduced the referral time and helped in early treatment and management of AES cases. The AES admissions increased at ETC centres to 60% and overall case fatality rate of AES declined to 3%. Under clean India mission and Jal Jeevan mission, proportion of population with clean drinking water increased from 74.3% in 1992 to 98.7% in 2020. The proportion of household having toilet facilities increased from 22.9% in 1992 to 67.4% in 2020. Provisions for better nutritional status under state and national nutrition mission helped in reducing the burden of stunting (52%) and wasting (53.4%) among under five children in 1992 to 38.8% (stunting) and 36.8% (wasting) in year 2018. These factors have all likely contributed to steady AES decline observed in UP.

**Conclusion:**

There is a recent steady decline in AES incidence and CFR since implementation of intensive AES surveillance system and JE immunization campaigns which is highlighting the success of interventions made by central and state government to control seasonal AES outbreaks in UP. Currently, AES incidence is 9 cases per million population (in year 2019) and mortality is 5.8%.

## Introductory Overview of Acute Encephalitis Syndrome (AES) Outbreaks in Uttar Pradesh, India

Acute encephalitis syndrome (AES) is a serious public health problem in India (1). India is a second-most populous country with 1.2 billion populations and comprises 28 states and 8 union territories (2). In India, first AES case was clinically diagnosed in the state Tamil Nadu in the year 1955 (3). Until 1973, AES outbreaks were confined to South India, with low prevalence (3). Later, AES outbreaks spread to other parts of India and now endemic in 171 districts of 19 states of India including Uttar Pradesh (UP) (3). UP is the most populous state of India divided into 18 divisions and 75 districts with a population of 199.8 million people (census 2011) (4). About 86% of total AES cases are from eastern part of UP (5) which reported mainly from Gorakhpur, Kushinagar, Maharajganj, Deoria, Siddharth Nagar, Sant Kabir Nagar, Basti and two adjoining districts of Bihar (Gopalganj and West Champaran) (5).

The mortality rate of AES in UP ranges between 8 and 35% (3), and those affected are typically the children aged <15 years and young adults (6). The AES patients usually present with acute onset of fever and altered sensorium with rapidly worsening clinical condition leading to death within hours. Those survivors may suffer from long-term health consequences, as 30–40% survivors have had residual neurological sequelae leading to poor quality of life (3).

In UP, the first AES outbreak was occurred in 1978, which caused more than 3,500 cases and 1,100 deaths (mortality = 31.4%) (6). Between 1978 and 1987, UP reported 9,299 suspected JE/AES cases and 3,103 deaths (6). Afterwards, extensive and recurrent outbreaks occurred in the years 1988 and 2005 in the state (6). AES outbreak in 2005 was the most devastating epidemic in UP, which caused more than 5,000 cases and 1,300 deaths, respectively, followed by further massive outbreaks in 2006 and 2007 with more than 5,000 cases and 1,173 deaths (6). During the years 2008–2018, UP reported 36,509 AES cases and 5,700 deaths (5).

In 2008, term AES was coined by World Health Organization (WHO) for surveillance and proper reporting of suspected encephalitis cases in India (7). WHO defined an AES case as “*a person of any age, at any time of the year with an acute onset of fever and a change in mental status (including symptoms such as confusion, disorientation, coma, or inability to talk) and /or new onset of seizures (excluding simple febrile seizure)” (7)*. The causative agent of AES varies with season and geographical location (8), so regional data on epidemiology and etiology of AES outbreaks were required for the formulation and effective implementation of outbreak management strategies. Initially, *Japanese encephalitis virus (JEV)* was the leading known cause of these AES outbreaks, and symptomatic treatment was the mainstay (8). In 2006, mass immunization campaign against JE was launched in JE endemic regions of India (11 districts) in which children aged 1 to 15 years were immunized with single dose of SA-14-14-2 live attenuated Chinese vaccine (9). In 2007, children from 27 highly JE endemic districts of India were immunized against JE, 22 districts in 2008, and 30 districts in 2009 (9). In 2011, single dose of SA-14-14-2 vaccine was introduced in routine immunization under Universal Immunization Programme (UIP) in the 181 JE endemic districts of India (9). Children aged 16 to 18 months were immunized with single dose of JE vaccine along with 1st booster dose of DTP vaccine (8). In 2013, another dose of JE vaccine was introduced in UIP in which children were immunized at the age of 9 months along with measles vaccine (8).

Strong surveillance system is an integral part of any disease control programme (3). Initially, in the year 1978, JE surveillance was started in India under National Malaria Elimination Programme (NMEP) (6). Later in 2006, a national level AES surveillance guideline was developed by National Vector-Borne Disease Control Programme (NVBDCP) for reporting of AES cases and suspected or confirmed JE cases as per standard case definition (NVBDCP 2006) (7). Laboratory surveillance led to a finding of other non-JE AES etiologies such as *enterovirus, scrub typhus, dengue virus*, and other viral, bacterial, and Rickettsial aetiologies (10).

Various control programmes were implemented by state and central government to control JE vectors, piggeries management by its segregation from human habitat and JE immunization. However, the findings of other non-JE etiologies of AES shifted the control strategies in other sectors (10). The finding of *enterovirus* in AES samples of 2009 outbreak set programmes for better hygiene and safe drinking water in motion, and *Orientia tsutsugamushi* (scrub typhus) positivity in retrospective AES samples of 2015 outbreak led the formulation of empirical doxycycline or azithromycin drugs and its availability at peripheral health centers for the treatment of febrile illness cases (10). Another massive AES outbreak in 2017 magnified the inefficiencies of healthcare services and poor health infrastructure in eastern UP following which strengthening of health infrastructure took place at district and peripheral level by constructing Encephalitis treatment Centre and Paediatric Intensive Care Unit (PICU) at peripheral health centres, and 24 x 7 ambulance service “Dial 108” was started to take AES patients to the nearby health center on time (5).

Acute encephalitis syndrome surveillance was the second-most focussed area after laboratory diagnostic component, which helped, in better characterization of AES epidemiology, disease burden, distributions, trends, and risk factors to guide the interventional programmes (7). The surveillance includes identification of an AES based on the WHO case definition (7) and then the classification of the case according to laboratory findings. A suspected AES can be classified into four ways: laboratory confirmed case, probable cases, AES due to other agents (other than JE), and AES of unknown aetiology (11).

The Ministry of Health and Family Welfare in collaboration with five other ministerial departments of India have implemented a comprehensive public health approach in attempt to reduce the burden of illness due to AES outbreaks in UP (3). By taking these actions, the central and eastern UP government has reduced AES mortality from 33% (1980–1984) to 12.6% (2015–2019) and managed annual and seasonal outbreaks over the years. The aim of this study was to describe the AES outbreaks in UP during 1978–2020, and public health action was taken aiming to reduce the burden of associated illness. This was done by a thorough review of scientific literature and Indian government policy guideline analysis.

## Methodology

Acute encephalitis syndrome-related data were retrieved from original articles available on PubMed, Google Scholar, Cochrane, MEDLINE, and Research Gate published during 1978 and 2020. Keywords used were “Acute Encephalitis Syndrome,” “Japanese Encephalitis,” “Acute Encephalitis Syndrome surveillance in Uttar Pradesh,” “Control and prevention strategies for Acute Encephalitis Syndrome in Uttar Pradesh,” and “Epidemiology of acute encephalitis syndrome in Uttar Pradesh.” Annual progress reports available on websites of Indian Council of Medical Research (ICMR), National Vector-Borne Disease Control Programme (NVBDCP), and Integrated Disease Surveillance Programmes (IDSP) and also progress reports of several schemes by central and UP state health authorities were also reviewed to present in this article.

### AES Surveillance

The quality surveillance data were required for better understanding of epidemiology and etiology of AES. During 1978, after some of the first AES outbreaks, the surveillance was started under National Malaria Elimination Programme (NMEP) which described the epidemiology and etiology of AES cases (6). In the year 2003, NMEP was renamed as National Vector-Borne Disease Control Programme (NVBDCP) to deal with the prevention and control programmes of all vector-borne diseases (VBDs) prevalent in India including Japanese encephalitis and dengue (7). In 2005, intensified AES surveillance was started as a part of overall VBD surveillance especially focussing on laboratory investigations to characterize trends and burden of AES to guide programme-based interventions (7). In 2006, WHO recommended syndromic surveillance of AES cases started and Directorate of NVBDCP developed “*National Surveillance Guidelines for AES in India”* and advised all JE endemic states of India to report JE commonly under AES case definition (7). AES surveillance in India is focussed to generate authentic and reliable laboratory, epidemiological, clinical, and entomological data (7). AES surveillance mainly has four components:

#### Epidemiological Surveillance

The main purpose of AES epidemiological surveillance was to understand the disease burden, pattern, and trend. The knowledge of actual incidence of AES formed a basis for planning of control and prevention strategies. Epidemiological surveillance includes two components:

##### Laboratory-Based Serological Surveillance

Initially, JEV was the leading cause of acute encephalitis cases (12). Increase in non-JE cases with indistinguishable clinical signs and symptoms made JE diagnosis difficult for clinicians (12). Hence in 2006, WHO recommended syndromic surveillance of JE was initiated and case-reporting guidelines were issued to all JE endemic states of India with the advice of reporting JE commonly under AES case definition and defined an AES case as “*a person of any age, at any time of the year with an acute onset of fever and a change in mental status (including symptoms such as confusion, disorientation, coma, or inability to talk) and /or new onset of seizures (excluding simple febrile seizures) (12)*”. Suspected AES cases were classified into 4 groups, that is, laboratory confirmed JE, probable JE, non-JE AES, and unknown AES (13). For laboratory investigations, blood and cerebrospinal fluid (CSF) of suspected AES patients were collected for further testing by IgM-based ELISA for aetiological confirmation (14). For other aetiological investigations, PCR-based diagnostics are used (14). AES surveillance was carried out through sentinel sites with and without laboratory facilities at the block level and informer units (IUs) at village level (11). Simultaneous to identify the aetiological agents of AES, uniform clinical guidelines for differential diagnosis of JE and non-JE AES cases were developed for which detailed listing of clinical symptoms was carried out (15). In 2014, an expert group from ICMR recommended algorithm entitled “*Laboratory testing algorithm for AES in India*” which was successfully rolled out for investigation of other non-JE viral or bacterial causes of AES (11). Team of researchers from different ICMR institutes, Manipal Centre for Virus Research (MCVR, Manipal), Christian Medical College (CMC, Vellore), and Jawaharlal Institute of Postgraduate Medical Education and Research (JIPMER, Puducherry) investigated AES samples for other viral and bacterial aetiologies (10). In 2016, an “*AES Cell”* was established by ICMR at Pediatric Department of Baba Raghav Das Medical College (BRDMC), Gorakhpur, to centralize the procedures for the collection and storage of clinical specimens and medical records of AES patients and also laboratory testing (14). The findings of aetiological investigations were retrieved from annual progress reports of National Institute of Virology, Gorakhpur field unit (now Indian Council of Medical Research-Regional Medical Research Centre, Gorakhpur (ICMR-RMRC, Gorakhpur) and presented in this article.

##### Clinical Surveillance

All public and private hospitals and health institutions attending AES patients were suggested to look out for signs and symptoms of encephalitis among patients and to report suspected cases based on standard case definitions in a standardized AES reporting format to higher authorities (11).

#### Environmental Surveillance

AES etiologies such as JE, dengue, and scrub typhus have variable hosts and vectors, and their surveillance has to be multipronged approach. The meteorological factors such as temperature, humidity, and rainfall patterns affect the vector reproduction, population, density, and distribution (14). Hence, these factors used as alarm signal for AES outbreaks and also suggested the best time to implement vector control programmes (15). The findings of environmental surveillance were summarized to present in the article.

#### Entomological Surveillance

Understanding of vector behavior and impact of vector control programmes can be assessed by entomological surveillance, which includes identification and spatial distribution of v*ectors, m*onitoring *of ad*ult and larval *density*, breeding, f*eeding and resting behavior* of vectors, susceptibility and resistance to insecticides, and detection of viral activity into vectors (15). The entomological surveillance requires specialized skills and trained manpower for which entomology laboratory was established at the National Institute of Virology (NIV) Gorakhpur unit in eastern UP (15). The data on the incidence of VBDs in the region were analyzed to assess the impact of vector control programmes initiated in the region.

#### Veterinary Surveillance

JE and ST are the vector-borne zoonotic diseases with animals and birds as natural reservoirs. Veterinary surveillance is necessary to measure the prevalence and density of amplifier hosts, serological surveillance for viral activity in reservoir host. Pigs are amplifying host of Japanese encephalitis virus and unorganized piggeries were the major issue in the region (6). The investigation on seroconversion and seroprevalence of JE was done in animals including pigs, goats, buffalo, etc., (16). The major findings were reviewed to present in the article.

### Development of Diagnostic Kit and Therapeutics for AES Cases

Until December 2011, commercially available diagnostic kit developed by Panbio Inverness Medical, Australia, was used for JE diagnosis in India (17). Later, ICMR- NIV, Pune, developed a highly reliable IgM capture ELISA kit for rapid diagnosis of JEV in serum/CSF and this kit can efficiently capture IgM against both GI and GIII genotypes of JEV (18). This kit is routinely supplied all over India for JE diagnosis. The performance of this kit was evaluated by Christian Medical Centre, Vellore, Tamil Nadu, India (19) and Centre for Disease Control, USA (20). According to CDC, NIV kit has sensitivity of 75% (CSF) and 71% (serum) and also specificity of 96% (CSF) and 77% (serum) (20). The data of JE testing were retrieved from annual progress reports of NIV, Gorakhpur field unit during 2005 and 2020.

Since 2006, the Chinese JE vaccine SA-14-14-2 has been used in JE immunization campaigns of India. In 2015, NIV Pune and Bharat Biotech developed first indigenous JE vaccine “JENVAC” from Indian isolate Kolar-821564XY JEV strain (21, 22). As per the recommendation from Indian Academy of Pediatrics (IAP) (2018-19 meet), the JENVAC vaccine should be administered at 12 months (first dose) and 13 months (booster dose) (23). In 2015, scrub typhus observed as the leading cause of AES in UP following which expert group from ICMR recommended intravenous azithromycin to all AES patients in scrub typhus endemic area (24). However, case fatality rate (CFR) was continued to be high, that is, 14.1% among treated patients compared to 17.7% untreated patients indicating low response to intravenous azithromycin treatment after advanced disease with CNS involvement (24). ICMR reconsidered the treatment protocol and recommended state health authorities to administer empirical doxycycline or azithromycin (EDA) to children with acute undifferentiated febrile illness (AUFIs). A guideline for the management of scrub typhus was launched by ICMR, and all febrile cases were recommended to provide presumptive EDA treatment at primary health centres of India (25). In 2016, the Government of UP issued a guideline to all health facilities of Gorakhpur division to follow this presumptive treatment protocol among febrile cases (26). The data of scrub typhus cases that occurred in the region were analyzed to assess the impact of EDA treatment among febrile cases.

### Healthcare Services and Laboratory Facilities

India is a country with mixed healthcare systems which include both public and private healthcare service providers (27). Due to federal system of governance in India, health system has been divided between union and state government (25). UP is the most populous state of India, home to over 200 million people (28). UP usually ranked lowest in health- and mortality-related indicators. For instance, in the recent report of National Health Index 2017–2018 by NITI AYOG, UP ranked at the bottom in the healthcare improvement (29). Following the massive annual AES outbreaks in eastern UP during 1978–2007, ICMR-NIV, Pune, established its field unit in Gorakhpur district of eastern UP in 2008 (30). This unit was solely responsible for AES diagnosis, patient's sample storage, outbreak investigations, and related research. The efforts to improve healthcare resources in medical colleges of UP were started early in 2005. In BRD Medical College of Gorakhpur, first ventilator equipped six beds intensive care unit (ICU) was established in 2005. Later, an epidemic ward with 10 more ICU beds was sanctioned in 2009 (31). In 2013, hundred-bed encephalitis ward was established in BRDMC (32). Following the outbreaks during 2017, state government focussed on strengthening health infrastructures in the region by constructing well-equipped 10 beds PICU at district hospitals, Encephalitis Treatment Centres (ETC) at community health blocks, availability of 24^*^7 ambulance services (Dial 108), and active fever-tracking system at peripheral health centres (33). Initially, during 2011, a rehabilitation specialist trained in assessment, quantification, and management of disabilities due to AES was hired in government medical colleges of UP (BRD Medical College) (11). Further, in 2018, a separate department of Physical Medicine and Rehabilitation (Child Rehabilitation Centre) was established in the campus of BRD Medical College, Gorakhpur, to cater the needs of disabled patients (34). At present, three PMR centres are functional in UP (35). The impact of these healthcare services on AES cases were analyzed to present in the article.

### Strengthening and Expansion of JE Vaccination

Because JEV involves pigs and mosquitoes in its transmission cycle, JEV elimination from the natural cycle is impossible. In April 2008, an expert committee meeting was held at ICMR headquarters on the recommendation of National Technical Advisory Group on Immunization (NTAGI) (23). In this meeting, postmarketing surveillance (PMS) studies for JE vaccine efficacy was recommended, and an unpublished study by ICMR revealed that the protective efficacy of the SA-14-14-2 live attenuated JE vaccine in India is not as high as that seen in Nepal (23). JE vaccination in India was started in 2006 in highly JE endemic districts, including 7 districts of UP (9). Large-scale mass immunization campaigns were carried out to immunize children between 1 and 15 years of age with a single dose of Chinese SA-14-14-2 vaccine (23). In 2011, JE immunization was introduced into UIP, and children aged 16 to 18 months get immunized against JE with a single dose of Chinese SA-14-14-2 vaccine (23). Later in 2013, another dose of SA-14-14-2 as booster dose to children at 9 months of age was introduced (36). In the year 2012–2013, ICMR-National Institute of Epidemiology, Chennai and ICMR-NIV Gorakhpur unit conducted surveys to estimate JE vaccine coverage in seven districts (Gorakhpur, Deoria, Kushinagar, Maharajganj, Kheri, Sant Kabir Nagar, and Siddharthnagar) of the Gorakhpur and Basti division and found low vaccine coverage (coverage for single dose and for two doses in Gorakhpur division was only 72.3 and 42.3%) and also identified unawareness and misconceptions of JE vaccination as a major factor attributable to JE vaccination failure (37). Immunization division of MoHFW in coordination with health authorities of UP has implemented programmes for the strengthening and expansion of JE vaccination for which “*Mission Indradhanush*” was launched in 2013 and UP state government with PATH foundation helped in scaling up of JE immunization coverage in the region (36). The status of JE vaccination coverage and the impact of vaccination on JE incidence were analyzed to present in the article.

### Effective Communication, Awareness Programmes, and Behavior Change

Major hurdles in the successful implementation of AES control and prevention programmes have been observed over the years, including unawareness of disease among general public, misinformation or no information about disease, and general vaccine hesitancy (37). To assess the level of hygiene in the community, we used access to toilets as an indicator and used National Family Health Survey (NFHS) reports from 1995 to 2019. To improve hygiene, Government of India addressed this issue by developing a prototype in Hindi for creating awareness among the general public residing in most affected region of UP and trained community volunteers for disseminating information to the community through these IEC materials (11). Under “*clean India mission (Swaccha Bharat Abhiyan),”* launched in 2014, people made aware of good hygiene and sanitation practices, clean drinking water, and avoiding open defecation (38). In 2019, Government of India launched “*Jal Jeevan Mission*” to provide clean tap water to every household in AES-affected areas (38). In 2018, UNICEF and UP state government jointly started “*Dastak campaign,”* which was a part of Comprehensive Social and Behavior Change Communication (SBCC) programme in which door-to-door awareness about JE, AES, and other communicable disease was given by trained community health workers, accredited social health activist (ASHA) or auxiliary nurse midwife (ANM), and Anganwadi (39). To assess the impact of these behavior change programmes, we used clean drinking water and toilet facilities as indicators. The related data were retrieved from UNICEF-Multiple Indicator Cluster Surveys (MICS) and National Family Health Survey (NFHS) during 1992 and 2020 to present in the article. The annual cases of water-borne diseases in UP were retrieved from National Health Profile Report of Government of India to determine the impact of these programmes on water-borne diseases.

### Improving Nutritional Status of Children

Malnutrition is a major problem not only in UP but in whole India and worldwide (40). UP is the most populous state with a population of 40% of all Indian population (28). According to the State Nutrition Mission fact sheet, in UP, half of all under 5 children are stunted and 10% are wasted (41). The main objective of SNM is to improve nutrition programmes across different sectors including Integrated Child Development Services (ICDS) and the National Health Mission (NHM) (41). To assess the nutritional status of children, we used the NFHS data during 1992 and 2016 and comprehensive national nutrition report (CNNR) from 2016 to 2018. We assessed malnutrition indicators such as stunting, wasting, and underweight. We aimed to assess whether major interventions had an impact to nutritional status of children and therefore likely reducing AES morbidity and mortality. During the study period, UP state government launched “*State Nutrition Mission (SNM)”* having UNICEF as its technical partner in 2014 (41). In 2018, Government of India launched “*Poshan Abhiyaan”* (*National Nutrition Mission*) to improve nutritional outcomes for children, adolescent, and pregnant and lactating mothers by targeted approach and convergence (42).

## Results

**Figure 1:**
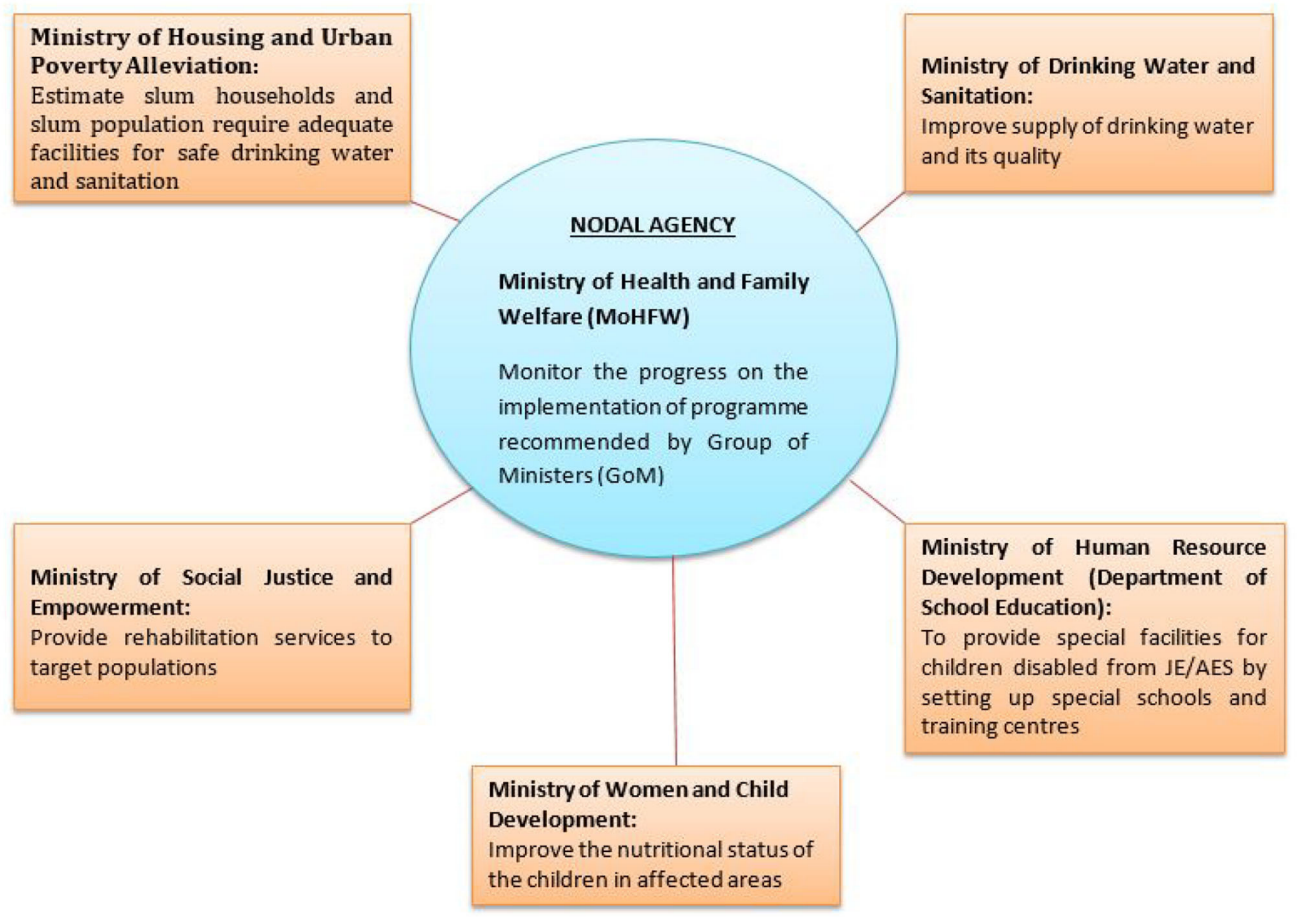
Convergence of five ministries/departments to resolve complexities involved in the implementation of national programme for prevention and control of JE/AES.

Uttar Pradesh is the highly AES endemic state of India since 1978 and contributing largely to the national burden of AES cases and mortality (6). Since first AES outbreak, the control measures were an emergency affair, which could only be done by active cooperation of different ministerial and non-ministerial bodies. The Ministry of Health and Family Welfare, Government of India (MoHFW) in collaboration with five other ministries developed a multipronged strategy through interdepartmental consultation to prevent AES outbreaks in India (7) (Figure 1). The major challenges and critical issues addressed to develop effective interventional programmes and public health policies to control AES outbreaks in UP and their impacts are as follows:

### Findings of AES Surveillance

#### Findings of Epidemiological Surveillance of AES Cases

**Figure 2:**
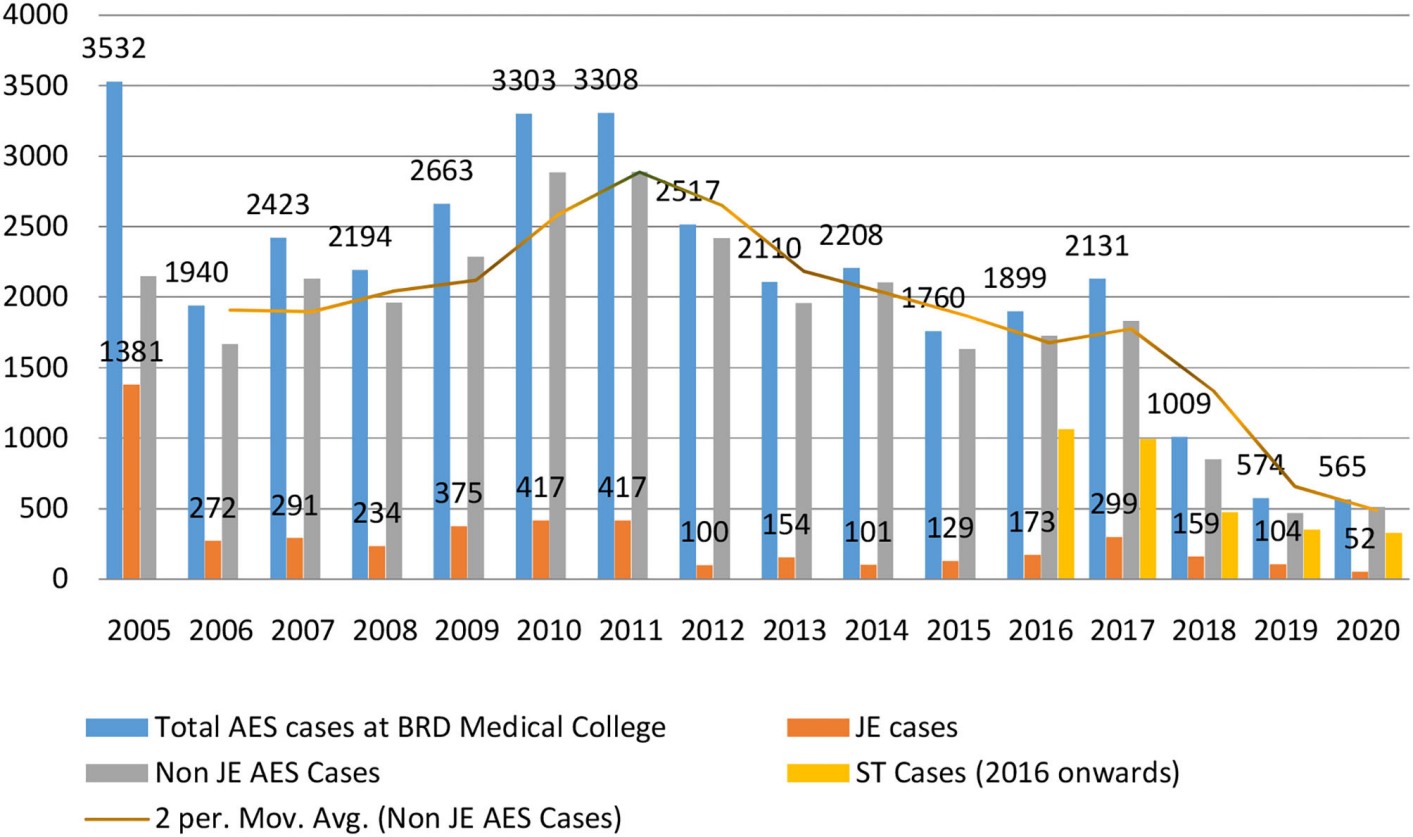
Decline in burden of AES cases due to Japanese encephalitis (JE), non-JE AES, and scrub typhus at BRD medical college, Gorakhpur.

First suspected JE outbreak (viral encephalitis then) in UP caused 3,550 cases (incidence = 34 cases/million population) and 1,117 deaths (CFR = 31.5%) in the year 1978 Supplementary Material 1. As observed in Table 1, during 1980 and 1984, case incidence was 4 cases/ million populations which increased to 18 cases/million population during 2005 and 2009. Afterwards slight decline in incidence (17 cases/million population) observed during 2010 and 2014. During 2015–19, incidence declined to 15 cases/million population [CI 12.6–20.6, *P*-value < 0.001]. As observed in Supplementary Material 1, since the implementation of intensive AES surveillance programmes and JE immunization campaigns during 2005 and 06, AES incidence declined to 9 cases/million population in 2019 from 31 cases/million population in 2005, and CFR declined to 5.8% in 2019 from 24.8% in 2005. Decline in CFR can be observed from 33% during 1980–1984 to 12.6% during 2015–2019 [CI 17.4–30.98, *p-*value < 0.001]. JE mass vaccination campaigns caused decline in JE cases from 38.8% in 2005 to 6% in 2020 (Figure 2). Whereas, JE cases declined in the region, the burden of non-JE AES remained high which led to the development of other control programmes and its implementation in the region. 2017 AES outbreak in UP caused 4,724 cases (AES incidence = 21 cases/million population) and 654 deaths (CFR = 13.8%) (Supplementary Material 1), and main etiology was scrub typhus (47.2%) (Figure 2). During 2018 and 2020, a sharp decline in AES cases can be observed which is mainly due to decline in non-JE AES cases including scrub typhus.

**Table 1:** AES cases, deaths, CFR, and disease incidence in UP during 1980–2019.

**Year**	**AES cases**	**AES deaths**	**AES Incidence per million population**	**Case fatality rate (CFR)**
1980–84	2,060	678	3.6	33
1985–89	5,565	1,908	9	34
1990–94	3,043	997	4.5	33
1995–99	3,584	748	4.7	20.8
2000–04	4,889	1,056	5.8	21.6
2005–09	17,010	3,653	18.2	21.5
2010–14	16,941	2,866	16.7	16.9
2015–19	16,802	2,110	15	12.6

**Figure 3:**
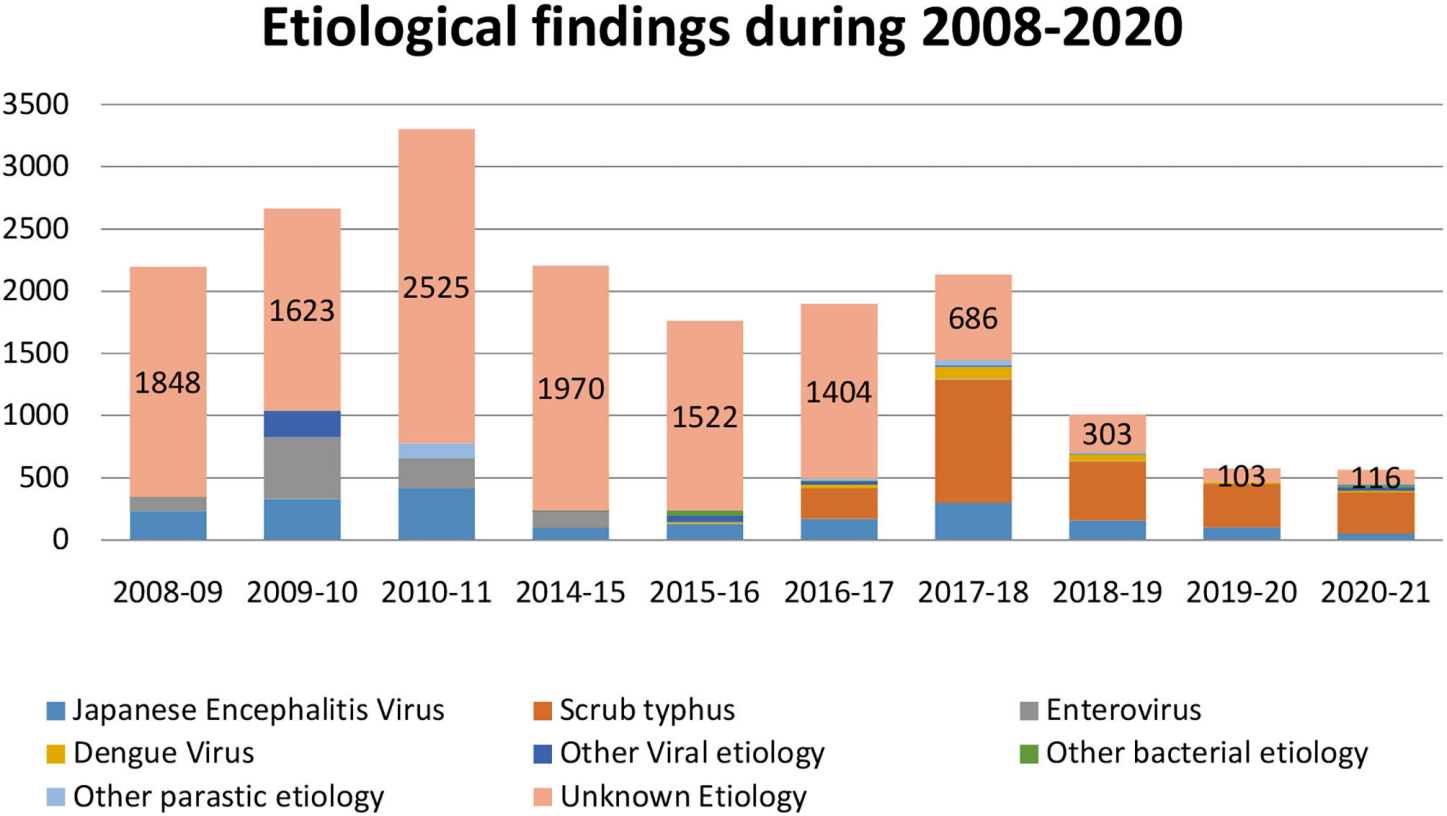
Graphical representation of aetiological investigations of AES cases during 1978–2020. In the year 2004, ICMR- NIV, Pune, started encephalitis outbreak investigations in Gorakhpur district of eastern UP, India. In 2008, field unit of ICMR-NIV, Pune, established in campus of Baba Raghav Das Medical College, Gorakhpur, for routine AES diagnosis and research. Decline in burden of unexplained AES cases can be observed.

During 2004 outbreak, investigations were conducted on 56 representative samples for the presence of JEV IgM antibody, and 20 samples (35.7%) confirmed positive for JEV. In the year 2005, 65 representative samples were investigated for JEV and 42 cases (64.6%) tested positive for JEV IgM antibody. During 2006 outbreak, JEV testing was done on 306 representative samples and 40 samples (13.1%) tested positive. Samples were also tested for the presence of *enterovirus (EV)* and 66 samples (21.6%) tested positive for EV RNA. In the year 2007, 663 representative samples tested for JE IgM and 488 representative samples tested for EV RNA. A total of 120 samples (18%) tested positive for JEV and 10 samples (2%) confirmed for EV etiology. After establishment of NIV field unit at Gorakhpur in July 2008, routine diagnosis of all AES samples was started. Samples were collected from 2,194 cases, and 234 cases (10.7%) were tested positive for JE IgM whereas 112 cases (5%) tested positive for EV RNA. Earlier, in majority of cases, etiology was not known for which extensive investigations for other possible etiologies were conducted during the further years. During 2004–2020, 12,901 CSF, 14,321 serum, 1,057 throat swabs, 3,325 rectal swabs, 5 brain biopsies, and 1,366 stool samples were collected for aetiological investigations. The epidemiological, clinical, and biochemical parameter investigations of each case led to the development of diagnostic algorithm for JE negative cases. As shown in Figure 3, the aetiological investigations led to the better understanding of AES etiologies. The burden of unexplained or unknown AES decreased from 1,848/2,194 (84.2%) in 2008 to 116/565 (20.5%) in 2020. The other viral, bacterial, and parasitic etiologies detected in AES samples during 2008–2020 are *hepatitis A/E, herpes groups, varicella zoster virus, Epstein barr virus, flavivirus generic, measles, mumps, parvovirus B19/P4, chikungunya virus, parainfluenza virus, malaria, other Rickettsials, Sporadic Neurocystisarcosis, Leptospira, M. tuberculosis, H. influenza, S pneumoniae, tubercular meningitis, Klebsiellaspp, Staphylococcus aureus, Entercoccusspp, Acinetobacterspp, Citrobacterspp, gram-positive cocci, Candida spp, and Salmonella typhi*.

#### Findings of Environmental Surveillance

Environmental surveillance studies carried out in the Gorakhpur region of eastern UP observed high prevalence o*f Culex quenquefasciatus* during winter and occurrence o*f Cx. tritaeniorhynchus* an*d Anopheles peditaeniatus* in transition season, that is, spring season. *Mansonia uniformis* showed its presence in March (43). Other studies suggested summer season as most suitable time for the introduction of intervention strategies for vector control due to very low circulation of the virus during the period (43). High prevalence of scrub typhus vector, *Leptotrombidium delicense*, observed during the rainy season (July–October) coinciding with AES incidence peak (44).

#### Findings of Entomological Surveillance

**Figure 4:**
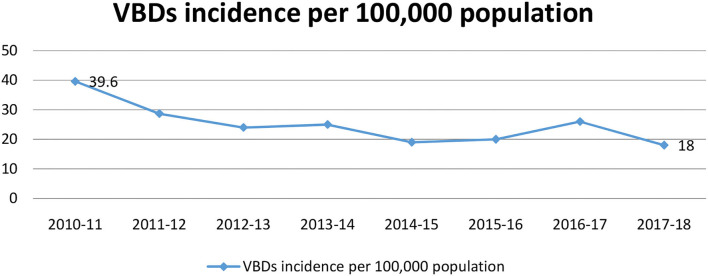
Impact assessment of vector control interventions and decline in burden of VBDs in UP.

Entomological and vector surveillance studies identified over sixteen mosquito species as JE vector belonging to genera o*f Anopheles* (3 spp)*, Culex* (10 spp), an*d Mansonia* (3 *spp) (**45**). Leptotrombidium delicense*, a Trombiculid mit*e*, was identified as a principal vector of scrub typhus (46). These mites reside in heavy scrub vegetation (45). The understanding of bionomics of JEV vector in the area helped in implementation of effective vector control programme in the region. Eastern UP is mainly a paddy growing area, with clay soil and a very high water table that favors the proliferation of immature stages o*f Cx. tritaeniorhynchus*, the principal vector for JEV (6). Thus, breeding control using larvivorous fis*h Gambusia affinis/Poecilia reticulate (guppy) in* all permanent water bodies before monsoon and paddy irrigation was recommended and initiated (6). These vectors are exophilic and endophagic in nature rest both indoor and mostly outdoor due to which vector control using indoor insecticide spray was technically not feasible and due to vast and enormous water bodies in the region antilarval strategies were resource intensive (12). Vector control by ultra-low volume (ULV) fogging was started targeting adult mosquitoes resting sites in the bushes, tree-shaded areas, and around domestic habitat (11). Vectors are most active in evening hours so fogging was recommended during the evening (11). Currently, Malathion and Pyrethrum formulations are used for fogging as recommended by NVBDCP (11). The decreased burden of VBDs is indicating the success of vector control programmes in the region. Data on the incidence of major VBDs that include malaria, kala azar, dengue, and JE were assessed. In Figure 4, a declining curve of incidence of VBDs can be observed from 39.6/1,00,000 population in 2010–2011 to 18/1,00,000 population in 2017–2018.

#### Findings of Veterinary Surveillance

Veterinary surveillance found JE seropositivity of 55–60% among pigs during JE non-transmission season (March–May) whereas 100% seropositivity during July–October (16). Shrew mous*e Suncus murinus* usually found in human dwellings observed as a vector fo*r Orientia tsutsugamushi* that causes scrub typhus in humans (46).

### Diagnostic Kit and Therapeutics for AES Cases

JENVAC reportedly has the highest geometric mean titer (GMT) response and long-term persisting neutralizing antibodies as compared to SA 14-14-2 Chinese JE vaccine in healthy children aged 1–15 years and found safe and efficacious for the age group between 1 and 50 years in Indian population (47). A pilot study in Gorakhpur was conducted to estimate the effectiveness of presumptive EDA treatment among febrile cases and its estimated effectiveness was 79.9^%^ (48). Decline in number of ST cases can be observed in Figure 2, which indicates the effective response of antibiotic therapy among febrile cases at peripheral health centers.

### Impact of Improvement in Healthcare Services and Laboratory Facilities

**Figure 5:**
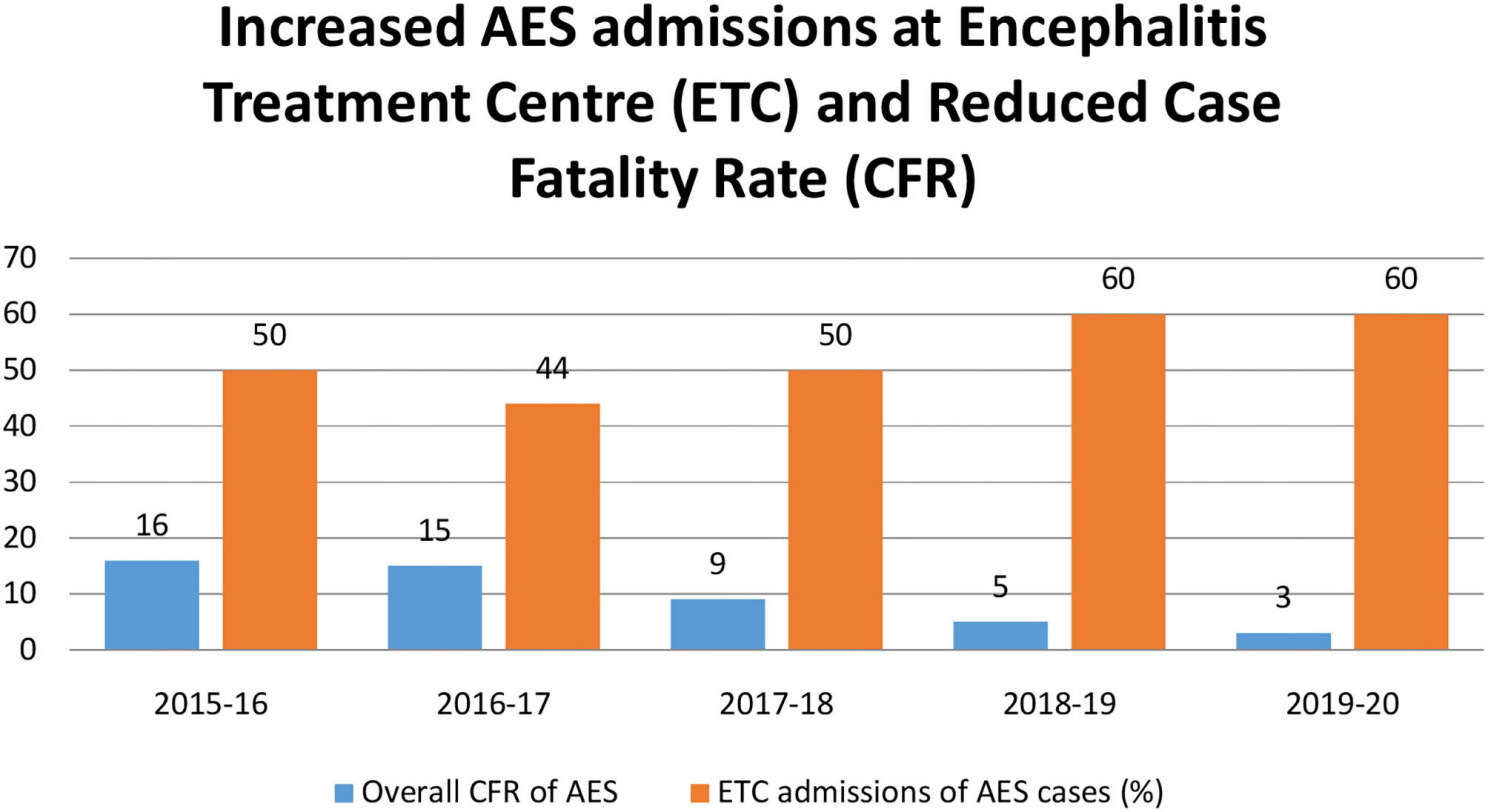
Graphical representation of increased AES admissions at Encephalitis Treatment Centre (ETC) constructed at peripheral health centers in UP and reduced CFR due to AES in UP during 2015 and 2020.

The changes in infrastructure increased AES admissions at ETC centres from 50% in 2016 to 60% in 2020, and patient was able to get their early treatment before any critical complications which might cause a decline in CFR among AES cases from 16% in 2016 to 3% in 2020 (Figure 5). As residual neurological sequelae in 30–40% of JE patients are observed, they made rehabilitation as an integral subspecialty in health system (11).

### Impact of Strengthening of JE Immunization

During 2006 mass vaccination campaign, 68,38,380 children aged 1–15 years from 7 JE endemic districts (Gorakhpur, Deoria, Kushinagar, Maharjganj, Kheri, Sant Kabir Nagar, and Siddharthnagar) of UP were targeted. Out of which 68,36,506 were vaccinated against JE with single dose of SA-14-14-2 live attenuated Chinese vaccine (99.97% vaccination coverage) (6). During 2007–2009, another 27 JE-endemic districts of UP were targeted for JE immunization in which 28,038,629 children aged 1–15 years get immunized against JE (6). Vaccination coverage was 96.74, 95.82, and 84.58% in the year 2007, 2008, and 2009, respectively (6). During the 2012–2013, a survey on JE vaccine coverage found low overall vaccine coverage (72.3% for single dose and 42.3% for booster/second dose) in Gorakhpur division (4 districts i.e., Deoria, Kushinagar, Gorakhpur, Maharajganj) (37). Decline in JE cases can be observed in Figure 2.

### Impact of AES Disease Awareness Campaigns and Behavior Change

**Figure 6:**
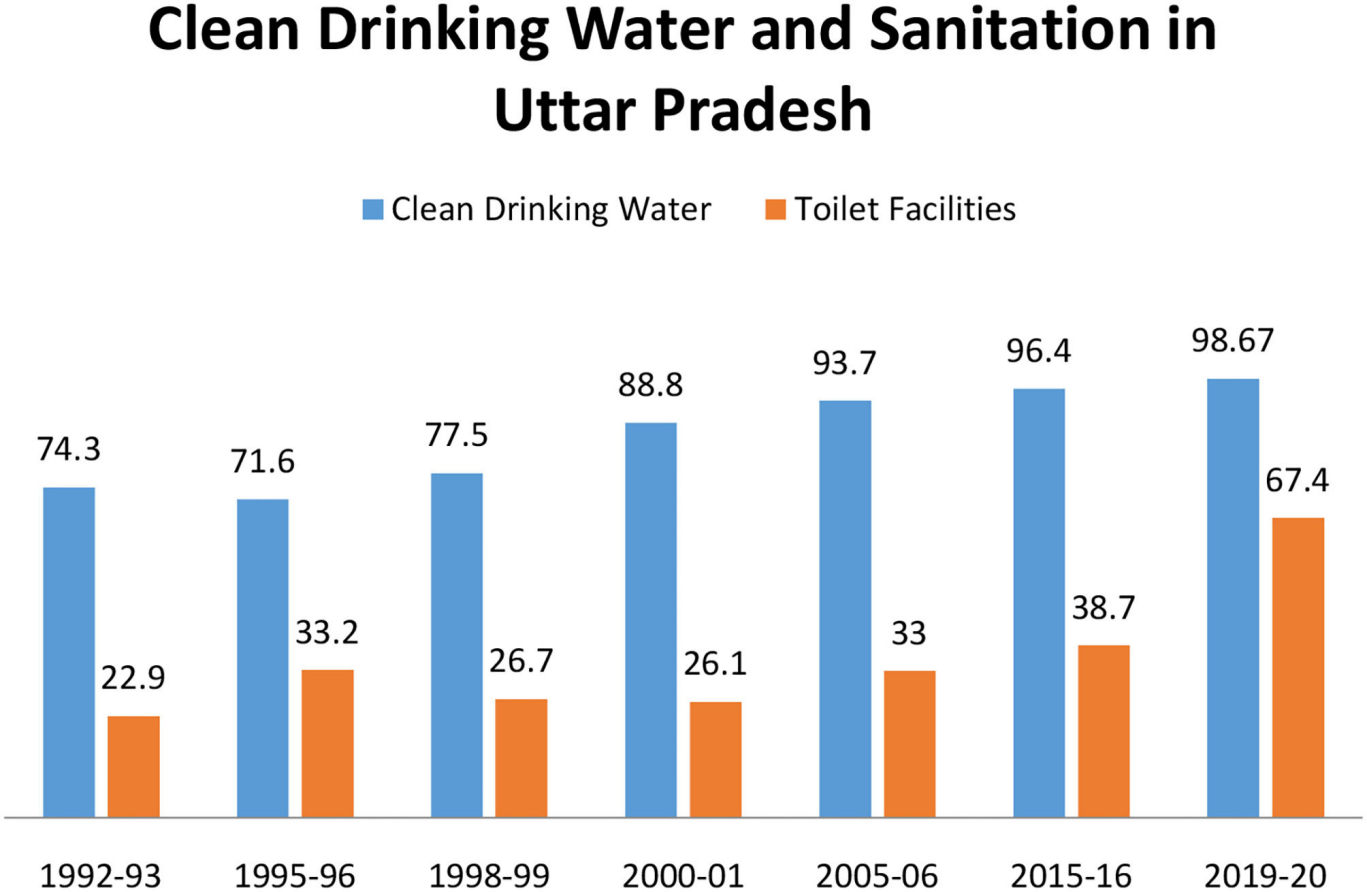
Graphical representation of status of clean drinking water and sanitation facilities in UP during 1992 and 2020. From the graph, it is evident that availability of clean drinking water and sanitation facilities has improved in the state in the past few years.

According to National Family Health Survey 1 (NHFS 1) during 1992–1993, only 74.3% household had access to clean drinking water and 22.9% household in UP had access to toilet facilities (Figure 6). During 2000–2001, UNICEF-MICS data of UP that showed increase in percent (88.8%) household had access to improved drinking water and 26.1% had toilet facilities. National Family Health Survey 3, during 2015–2016, showed that 96.4% people have access to clean drinking water and 38.7% people have access to toilet facilities. In recent survey, under Jalshakti mission by department of drinking water and sanitation of Government of India reported that 67.4% household in UP had access to toilet facilities and 98.67% household had access to clean drinking water (Figure 6). These data from NFHS and UNICEF-MICS suggest the positive impact of mass sensitization programme under clean India Mission, Jal Jeevan Mission, and Dastak Campaign. Poor sanitation and contaminated drinking water play a major role in transmission of diseases such as diarrhea, cholera, and typhoid. However, sanitation and clean drinking water facilities improved in UP but the burden of these diseases increased in the region (Data of National Health Profile, Government of India). In 2015, UP reported total 1,095,274 cases of diarrhea, cholera, and typhoid whereas in 2018, the reported cases were 2,072,508 (+89.2% rise) (49).

### Improved Nutritional Status of Children

**Figure 7:**
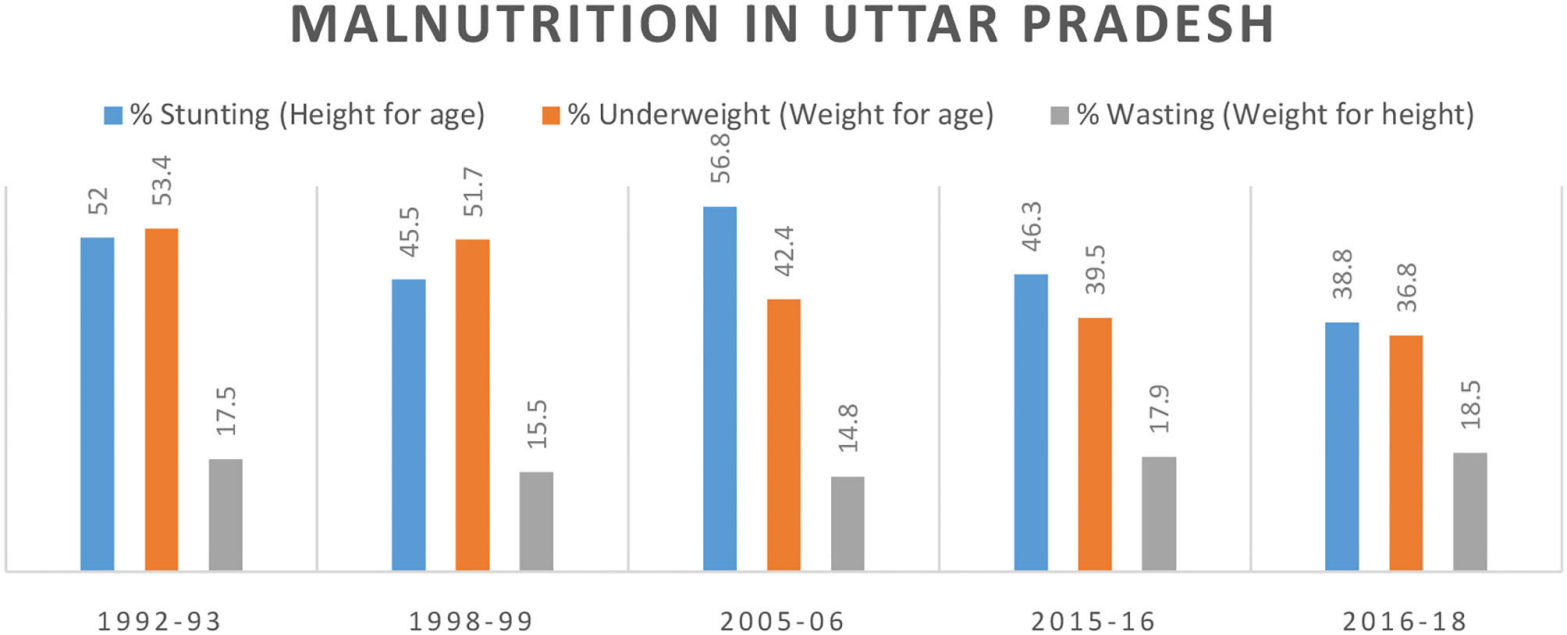
Reduced burden of malnutrition in UP highlights success of interventions implemented for better nutritional status of children.

The SNM and NNM were started in the year 2014 and 2018, respectively. According to NFHS 1 data, during 1992–1993, about 52% of under five children developed stunting, 53.4% were underweight, and 17.5% developed wasting (Figure 7). NFHS round 2 took place during 1998–1999 in which decline in stunting (45.5%), underweight (51.7%), and wasting (15.5%) was observed in under five children in UP. Until 2005, stunting remained on a high level of around 50%, but has declined since to 38.8% (<40%) in 2016–2018 CNNS survey (Figure 7). The underweight, a more acute measure, started to decline earlier around the year 2000. This reduced burden of malnutrition in the region is highlighting the impact of SNM and NNM programme in a positive way.

## Conclusion and Recommendations

The lack of surveillance data and laboratory testing capacity was one of the major challenges during AES outbreaks in the region that was tackled by developing a robust surveillance system, establishment of NIV Gorakhpur unit, and AES cell in BRDMC. A systematic diagnostic algorithm implemented for AES aetiological investigations helped in developing specific therapeutics and treatment protocols for AES etiologies in the region. Aggressive JE immunization campaigns and the use of presumptive treatment of azithromycin or doxycycline for febrile illness helped in managing and reducing the JE and ST cases which caused overall decline in AES cases. The surveillance studies identified the risk factors for AES etiologies and helped in planning appropriate interventions that caused direct or indirect impact on number of AES cases in the region. The *Culex vishnui* subgroup is recognized as a major vector of JEV and play an important role in disease epidemiology. However, virus may be present in other mosquitoes but their vectorial capacity has to be established further.

Another major challenge was differential diagnosis due to high incidence of mimickers such as pyogenic meningitis, cerebral malaria, and tubercular meningitis, acute disseminated encephalomyelitis as these required prompt additional specific treatments for which AES case definitions, diagnostic guidelines, and algorithms specific to AES were developed.

Poor health infrastructure at the tertiary and peripheral level, inappropriate response, and lack of knowledge were the major roadblocks to reduce the AES mortality largely. To combat this, health infrastructure was strengthened at tertiary and peripheral level by establishing pediatric wards and ICUs with trained staff with all set of required equipment that helped patient treatment quickly. A rapid response team was formed at hospitals trained to handle the crisis. A uniform case-reporting format and guidelines were developed by NVBDCP. The mass sensitization programmes of UP government such as *Dastak* and *Sanchari Rog Niyantran Abhiyan (SRNA)* supplements the central government's multipronged approach to reduce the case fatality due to AES. The increase in burden of water-borne diseases by 89.2% in the region suggests that clean India Mission, Jal Jeevan Mission, and Dastak Campaigns have a long way to go for assuring the clean drinking water supply, sanitation, and education of the mass till the village levels.

A significant fall observed in the burden of malnutrition in the region highlighting the success of various interventions implemented such as “*State Nutrition Mission*” and “*Poshan Abhiyaan*” to improve the nutritional status of children in UP. Over the years, due to joint efforts of central and state government agencies, the AES situation was improved significantly. The decade's long war with JE/AES is now on its way to see the final assault as evidenced by decline in AES cases by 70% and mortality by 90% in 2019. “*United we stand*” strategy worked successfully in which 11 departments or ministries came together to fight against this annual scourge and multipronged strategy against multifaceted problem and also microplanning by each department helped in achieving the desired results. The major interventions implemented in the region affected the number of AES admissions and decline in AES cases observed over the years depicts the success of these interventions. A timely gap analysis by realizing overburden of AES cases on BRD Medical College and steps taken by investing in improving the health infrastructures of peripheral health blocks and district hospitals made case management easy at peripheral level and caused reduction in CFR. The history has taught us a lesson, any complacency for any disease after its control or /management hits back with more vengeance; hence, the achievements gained need to be maintained and cemented further with more collaborative efforts to achieve victory on this greatest misery to humankind.

## The Way Forward

Eastern UP has successfully controlled the AES outbreaks; now, a regional strategy and action plan are required to take AES graph to zero level. JE is a vaccine-preventable disease, in spite of several control measures took place, sporadic cases still observed in eastern UP. Existing programmes and interventions are feasible to eliminate JE in the region. A coherent and coordinated approach is required to increase the effectiveness and efficiency of programmes by identifying and maximizing the synergies between the programmes. Integrated people-centred healthcare approaches need to be established which include people and healthcare service delivery units. Surveillance data on JE vaccine efficacy, sustainability, and interchangeability will be critical to inform new vaccination policies. Most of the identified etiologies for AES are treatable and therapeutics is available for them. AES with unknown etiology still contributes to 15–20% of total AES cases in the region. High-throughput DNA sequencing technologies may offer unprecedented opportunities to detect unrecognized or novel pathogen for unexplained AES. Possibilities of non-infectious cause, that is, autoimmune encephalitis should also be explored in unknown AES cases. Efforts should not focus on vaccination alone but also on other aspects of preventive measures which include integrated vector control measures, active fever-tracking system, early referral, community awareness, and case-based surveillance data.

## Author Contributions

The conception, design of manuscript, and drafting of article was carried out by NS, HD, and RK. Acquisition of data was performed by NS and HD. Analysis and interpretation of data was performed by NS. Critical revision of article for important intellectual content was carried out by RK, MM, VB, and HD. All authors approved the finial manuscript.

## Conflict of Interest

The authors declare that the research was conducted in the absence of any commercial or financial relationships that could be construed as a potential conflict of interest.

## Publisher's Note

All claims expressed in this article are solely those of the authors and do not necessarily represent those of their affiliated organizations, or those of the publisher, the editors and the reviewers. Any product that may be evaluated in this article, or claim that may be made by its manufacturer, is not guaranteed or endorsed by the publisher.

## References

[1] Granerod J Crowcroft NS. The epidemiology of acute encephalitis. NeuropsycholRehabil. (2007) 17:406–28.. 10.1080/09602010600989620 17676528

[2] States and Union Territories. Know India Programme. Available online at: https://knowindia.india.gov.in/ (accessed November 28, 2021).

[3] Narain JP Dhariwal AC MacIntyre CR. Acute encephalitis in India: an unfolding tragedy. Indian J Med Res. (2017) 145:584–7. 10.4103/ijmr.IJMR_409_17 28948947 PMC5644291

[4] Kopf D Varathan P. If Uttar Pradesh were a country. Quartz India (2017).

[5] Singh AK Kharya P Agarwal V Singh S Singh N Jain P. Japanese encephalitis in Uttar Pradesh, India: A situational analysis. J Family Med Prim Care. (2020) 9:3716–21. 10.4103/jfmpc.jfmpc_449_20 33102356 PMC7567188

[6] Kumari R Joshi PL. A review of Japanese encephalitis in Uttar Pradesh, India. WHO South East Asia J Public Health. (2012) 1:374–95. 10.4103/2224-3151.207040 28615603

[7] Guidelines for Surveillance of Acute Encephalitis Syndrome (With Special Reference To Japanese Encephalitis) NVBDCP. (2006). Available online at: http://www.nvbdcp.gov.in/Doc/AES%20guidelines.pdf (accessed December 12, 2020).

[8] National Health mission. Routine Immunization, Government of India. Available online at: http://www.nrhm.gov.in/nrhmcomponents/rmncha/immunization/background.html (accessed Janury 15, 2020).

[9] Vashishtha VM Ramachandran VG. Vaccination policy for Japanese encephalitis in India: tread with Caution! Indian Pediatr. (2015) 52:837–9. 10.1007/s13312-015-0728-5 26499003

[10] Murhekar M Vivian Thangaraj JW Mittal M Gupta N. Acute encephalitis syndrome in eastern Uttar Pradesh, India: changing etiological understanding. J Med Entomol. (2018) 55:523–6. 10.1093/jme/tjy042 29635529

[11] National Vector Borne Disease Control Programme (NVBDCP). National Programme for Prevention and Control of Japanese Encephalitis/Acute Encephalitis Syndrome –Operational guidelines. (2014). Available online at: https://nvbdcp.gov.in/Doc/JE-AES-Prevention-Control(NPPCJA).pdf [accessed July 15, 2020).

[12] WHO-Recommended Standards For Surveillance Of Selected Vaccine-Preventable Diseases. (2006). Available online at: http://www.path.or/ile/HOsurveillancestandardsJE.pdf [accessed October 1, 2020).

[13] *Clinical Management Of Acute Encephalitis Syndrome Including Japanese Encephalitis: Guidelines (NVBDCP)*. (2009). Available online at: https://nvbdcp.gov.in/Doc/Revised%20guidelines%20on%20AES_JE.pdf (accessed September 25, 2020).

[14] *Annual Annual Progress Report of National Institute of Virology Gorakhpur field Unit (2017-18)*. Available online at: https://rmrcgkp.icmr.org.in/images/pdf/AR%20GKP%20UNIT%202017-18.pdf (accessed September 17, 2020).

[15] Annual Annual Progress Report of National Institute of Virology Gorakhpur field Unit (2010-11) Available online at: https://niv.co.in/annualreports/AnnualReport1011/16GorakhpurUnit.pdf (accessed September 16, 2020).

[16] *Annual Annual Progress Report of National Institute of Virology Gorakhpur field Unit (2014-15)*. Available online at: https://niv.co.in/annualreports/AnnualReport1415/NIVGorkhpur%20Unit.pdf (accessed July 29, 2020).

[17] Jain P Singh AK Khan DN Pandey M Kumar R Garg R. Trend of Japanese encephalitis in Uttar Pradesh, India from 2011 to 2013. Epidemiol Infect. (2016) 144:363–70. 10.1017/S0950268815000928 26112391

[18] Kulkarni R Sapkal G Mahishi L Shil P Gore MM. Design and characterization of polytope construct with multiple B and TH epitopes of Japanese encephalitis virus. Virus Res. (2012) 166:77–86. 10.1016/j.virusres.2012.03.006 22445688

[19] Sathish N Manayani DJ Shankar V Abraham M Nithyanandam G Sridharan G. Comparison of IgM capture ELISA with a commercial rapid immunochromatographic card test & IgM microwell ELISA for the detection of antibodies to dengue viruses. Indian J Med Res. (2002) 115:31–6. 12138661

[20] World Health Organization. Regional Office for South-EastAsia. Fourth *Biregional Meeting on the Control of Japanese Encephalitis (JE). Report of the Meeting Bangkok, Thailand, 7–8 June 2009*. Available online at: http://www.wpro.who.in/mmunizatio/ocument/oc/EBiregionalMeetingJune2009final.pdf (accessed September 5, 2020).

[21] Kedarnath N Prasad SR Dandawate CN Koshy AA George S Ghosh SN. Isolation of Japanese encephalitis & West Nile viruses from peripheral blood of encephalitis patients. Indian J Med Res. (1984) 79:1–7. 6327509

[22] Singh A Mitra M Sampath G Venugopal P Rao J Krishnamurthy V. A japanese encephalitis vaccine from india induces durable and cross-protective immunity against temporally and spatially wide-ranging global field strains. J Infect Dis. (2015) 212:715–25. 10.1093/infdis/jiv023 25601942

[23] Vashishtha VM Choudhury P Kalra A Bose A Thacker N Yewale V. Indian Academy of Pediatrics (IAP) recommended immunization schedule for children aged 0 through 18 years-India, 2014 and updates on immunization. Indian Pediatr. (2014) 51:785–800. 10.1007/s13312-014-0504-y 25362009

[24] Murhekar MV Mittal M Prakash JA Pillai VM Mittal M Kumar CPG. Acute encephalitis syndrome in Gorakhpur, Uttar Pradesh, India - Role of scrub typhus. J Infect. (2016) 73:623–6. 10.1016/j.jinf.2016.08.014 27592263

[25] Rahi M Gupte MD Bhargava A Varghese GM Arora R. DHR-ICMR guidelines for diagnosis & management of Rickettsial diseases in India. Ind J Med Res. (2015) 141:417–22. 10.4103/0971-5916.159279 26112842 PMC4510721

[26] Government of Uttar Pradesh. AES/JE control strategy. Treatment with *Doxycycline/Azithromycin*. 21/F/S.NO/AES/JE/2016/2044-49.2016

[27] Choksi M Patil B Khanna R Neogi SB Sharma J Paul VK. Health systems in India. J Perinatol. (2016) 36:9–12. 10.1038/jp.2016.184 27924110 PMC5144115

[28] Government of India. Ministry of Home Affairs. Size, growth rate and distribution of population. In: Census of India. New Delhi: office of the Registrar General & Census Commissioner, India 2011. Available online at: http://censusindia.gov.in/2011-provresults/data_files/india/Fina_PPT_2011_vhapter3.pdf.

[29] Health Performance Report 2020- NITI Aayog. Available online at: https://www.niti.gov.in/sites/default/files/2020-02/Annual_Report_2019-20.pdf (accessed December 12, 2021).

[30] *Annual Annual Progress Report of National Institute of Virology Gorakhpur field Unit (2008-09)*. Available online at: https://niv.co.in/annual_reports/Annual_Report_2008_09.html (accessed July 14, 2020).

[31] National Rural Health Mission. State Action Plan. Uttar Pradesh. 2009-10. Available online at: http://upnrhm.gov.in/assets/site-files/pip2009-10.pdf (accessed December 20, 2021).

[32] National Rural Health Mission. 6^th^ Common Review Mission Report 2012. Available online at: http://nhm.gov.in/images/pdf/monitoring/crm/6thcrm/report/6th_CRM_Main_Report.pdf (accessed December 20, 2021).

[33] Operational Operational guidelines National programme for prevention control of Japanese encephalitis/ acute encephalitis syndrome NVBDCP. (2019). Available online at: http://www.nvbdcp.gov.in/WriteReadData/1892s/JEAES-PreventionControl(NPPCJA).pdf (accessed September 12, 2020).

[34] Setting up of District Disability Rehabilitation Centres in the Identified Districts. Available from: http://disabilityaffairs.gov.in/upload/uploadfiles/files/DDRC%20Scheme%20after%20SFC.pdf (accessed September 15, 2020).

[35] Annual progress report of Ministry of Health Family welfare 2020-2021. Available online at: https://main.mohfw.gov.in/sites/default/files/Annual%20Report%202020-21%20English.pdf. (accessed December 11, 2021).

[36] Report on Universal Immunization Programme. (2015). Available online at: https://main.mohfw.gov.in/sites/default/files/5628564789562315.pdf (accessed September 14, 2020).

[37] Murhekar MV Oak C Ranjan P. Coverage & missed opportunity for Japanese encephalitis vaccine, Gorakhpur division, Uttar Pradesh, India, 2015: implications for Japanese encephalitis control. Indian J Med Res. (2017) 145:63–9. 10.4103/ijmr.IJMR_712_16 28574016 PMC5460575

[38] Press Information Bureau (PIB). 97 Lakh Households Get Tap Water Supply In 5 Encephalitis Affected States In Just 22 Months. Available online at: http://pib.gov.in/PressReleasePage.aspx?PRID=1734397 (accessed July 10, 2021).

[39] *Dastak Campaign*. (2018). Available online at: https://gorakhpur.nic.in/scheme/dastak-campaign/ (accessed September 9, 2020).

[40] Gupta SK. Child Malnutrition in Uttar Pradesh: Inter district analysis. Int J Res analyt Rev. (2019) 6:8.

[41] Documentation of State Nutrition Mission in Uttar Pradesh. (2016). Available online at: https://r4d.org/wp-content/uploads/Documentation-of-SNM-UP.pdf (accessed March 14, 2020).

[42] *Transforming Nutrition in India: Poshan Abhiyaan Progress Report*. (2019). Available online at: https://www.niti.gov.in/sites/default/files/2020-02/.Poshan_Abhiyaan_2nd_Report_0.pdf (accessed January 22, 2021).

[43] Kanojia PC Shetty PS Geevarghese G. A long-term study on vector abundance & seasonal prevalence in relation to the occurrence of Japanese encephalitis in Gorakhpur district, Uttar Pradesh. Indian J Med Res. (2003) 117:104–10. 14575175

[44] Sadanandane C Elango A Panneer D Mary KA Kumar NP Paily KP. Seasonal abundance of Leptotrombidiumdeliense, the vector of scrub typhus, in areas reporting acute encephalitis syndrome in Gorakhpur district, Uttar Pradesh, India. Exp Appld Acarol. (2021) 84:795–808. 10.1007/s10493-021-00650-2 34328572

[45] Philip Samuel P Hiriyan J Gajanana A. Japanese encephalitis virus infection in mosquitoes and its epidemiological implications. Icmr Bull. (2000) (4):37–43.

[46] Sadanandane C Jambulingam P Paily KP Kumar NP Elango A Mary KA. Occurrence of Orientia tsutsugamushi, the etiological agent of scrub typhus in animal hosts and mite vectors in areas reporting human cases of acute encephalitis syndrome in the Gorakhpur Region of Uttar Pradesh, India. Vector Borne Zoonotic Dis. (2018) 18:539–47. 10.1089/vbz.2017.2246 30016222

[47] Vadrevu KM Potula V Khalatkar V Mahantshetty NS Shah A Ella R. Persistence of immune responses with an inactivated Japanese encephalitis single-dose vaccine, JENVAC and interchangeability with a live-attenuated vaccine. J Infect Dis. (2020) 222:1478–87. 10.1093/infdis/jiz672 31858116 PMC7529014

[48] Thangaraj JWV Zaman K Shete V Pandey AK Velusamy S Deoshatwar A. Effectiveness of presumptive treatment of acute febrile illness with doxycycline or azithromycin in preventing acute encephalitis syndrome in Gorakhpur, India: A cohort study. Indian Pediatr. (2020) 57:619–24. 10.1007/s13312-020-1889-4 32221056

[49] Report of National Health Profile (NHP) of India 2015-2018. Available online at http://www.cbhidghs.nic.in/showfile.php?lid=1147 (accessed December 12, 2020).

