# Supplementary material for: The Outbreaks of Acute Encephalitis Syndrome in Uttar Pradesh, India (1978–2020) and Its Effective Management: A Remarkable Public Health Success Story
Source: Front Public Health. 2022 Feb 9;9:793268. doi: 10.3389/fpubh.2021.793268 (PMC8863615; doi:10.3389/fpubh.2021.793268)
Supplement: Supplementary file 1 [file Table_1.DOCX]

**Supplementary Table:** AES cases, Deaths, CFR and Incidence during 1978-2020

| **Year** | **Population (10^6^)** | **AES cases** | **Deaths** | **AES Incidence per million population** | **CFR (AES)** |
| --- | --- | --- | --- | --- | --- |
| **1978** | 104 | 3550 | 1117 | 34 | 31.5 |
| **1979** | 106 | 150 | 48 | 1.4 | 32 |
| **1980** | 109 | 1604 | 530 | 14.8 | 33 |
| **1981** | 111 | 187 | 62 | 1.7 | 33 |
| **1982** | 113 | 118 | 28 | 1 | 23 |
| **1983** | 115 | 149 | 58 | 1.3 | 38.9 |
| **1984** | 117 | 2 | 0 | 0.02 | 0 |
| **1985** | 119 | 1187 | 409 | 9.95 | 34.5 |
| **1986** | 122 | 1855 | 626 | 15.3 | 33.7 |
| **1987** | 124 | 177 | 76 | 1.4 | 42.9 |
| **1988** | 126 | 772 | 249 | 6.1 | 32.2 |
| **1989** | 128 | 1574 | 548 | 12.3 | 34.8 |
| **1990** | 130 | 183 | 73 | 1.4 | 39.9 |
| **1991** | 132 | 1823 | 633 | 13.8 | 34.7 |
| **1992** | 136 | 768 | 224 | 5.7 | 29.2 |
| **1993** | 139 | 104 | 32 | 0.75 | 30.8 |
| **1994** | 142 | 165 | 35 | 1.2 | 21.2 |
| **1995** | 146 | 170 | 41 | 1.2 | 24.1 |
| **1996** | 149 | 672 | 161 | 4.5 | 24 |
| **1997** | 153 | 351 | 76 | 2.3 | 21.6 |
| **1998** | 156 | 1021 | 195 | 6.5 | 19.09 |
| **1999** | 159 | 1370 | 275 | 8.6 | 20.07 |
| **2000** | 163 | 1126 | 259 | 6.9 | 23 |
| **2001** | 166 | 1005 | 199 | 6.05 | 19.8 |
| **2002** | 170 | 604 | 133 | 3.6 | 22.02 |
| **2003** | 173 | 1124 | 237 | 6.5 | 21.08 |
| **2004** | 176 | 1030 | 228 | 5.8 | 22.14 |
| **2005** | 180 | 5581 | 1387 | 31.04 | 24.8 |
| **2006** | 183 | 2320 | 528 | 12.7 | 22.8 |
| **2007** | 187 | 3024 | 645 | 16.2 | 21.3 |
| **2008** | 190 | 3012 | 537 | 15.8 | 17.8 |
| **2009** | 194 | 3073 | 556 | 15.9 | 18.09 |
| **2010** | 188 | 3540 | 494 | 18.8 | 13.9 |
| **2011** | 200 | 3492 | 579 | 17.5 | 16.6 |
| **2012** | 204 | 3484 | 557 | 17 | 16 |
| **2013** | 207.6 | 3096 | 609 | 14.9 | 19.7 |
| **2014** | 212 | 3329 | 627 | 15.7 | 18.8 |
| **2015** | 216 | 2894 | 479 | 13 | 16.5 |
| **2016** | 220 | 3919 | 621 | 17.8 | 15.8 |
| **2017** | 224 | 4724 | 654 | 21.1 | 13.8 |
| **2018** | 228 | 3080 | 230 | 13.5 | 7.5 |
| **2019** | 232 | 2185 | 126 | 9.4 | 5.8 |
| **2020** | 237 | 1646 | 83 | 6.9 | 5 |

AES cases, deaths, CFR and disease incidence in Uttar Pradesh (*Data Source:* Data of acute encephalitis cases occurred during 1978-2020 were taken from both published and unpublished reports of state and central health department. Data on number of cases occurred during 1978-2005 were retrieved from Kumari R, Joshi PL. A review of Japanese encephalitis in Uttar Pradesh, India.*WHO South East Asia J Public Health*. 2012; **1**(4):374-395, NVBDCP annual reports and JE/AES case sheet and unpublished reports of state health department of UP) Available from: <https://nvbdcp.gov.in/WriteReadData/l892s/68457624441633502806.pdf> , <https://nvbdcp.gov.in/Doc/Annual-report-2011-12-NVBDCP.pdf> <https://nvbdcp.gov.in/Doc/Annual-report-NVBDCP-2014-15.pdf>

Data on population of state retrieved from CENSUS 1971, 1981, 1991, 2001, 2011.

Population data between 2011-2020 were retrieved from <https://www.indiaonlinepages.com/population/uttar-pradesh-population.html>
